# Hemodynamic consequences of intravenously given *E. coli* suspension: observations in a fulminant sepsis model in pigs, a descriptive case–control study

**DOI:** 10.1186/s40001-019-0372-y

**Published:** 2019-02-13

**Authors:** Mariann Berhés, Norbert Németh, Katalin Pető, Ádám Deák, Endre Hajdú, Ábel Molnár, Péter Árkosy, Judit Szabó, Béla Fülesdi

**Affiliations:** 1 Department of Anesthesiology and Intensive Care, Faculty of Medicine, University of Debrecen, Nagyerdei krt 98, Debrecen, 4032 Hungary; 2 Department of Operative Techniques and Surgical Research, Faculty of Medicine, University of Debrecen, Debrecen, Hungary; 3 Department of Oncology, Kenézy County Hospital, Debrecen, Hungary; 4 Department of Medical Microbiology, Faculty of Medicine, University of Debrecen, Debrecen, Hungary; 5 Outcomes Research Consortium, Cleveland, USA

**Keywords:** *E. coli*, Experimental sepsis, PiCCo monitoring

## Abstract

**Background:**

The aim of the present work was to assess systemic hemodynamic changes using PiCCo monitoring in a porcine model of *E. coli*-induced fulminant sepsis.

**Methods:**

Thirty-one healthy female Hungahib pigs were randomly assigned into control (*n* = 15) or septic groups (*n* = 16). In the *sepsis group Escherichia coli* culture was intravenously administrated in a continuously increasing manner according to the following protocol: 2 ml of bacterial culture suspended in physiological saline was injected in the first 30 min, then 4 ml of bacterial culture was administered within 30 min, followed by infusion of 32 ml bacterial culture for 2 h. Control animals received identical amount of saline infusion. Systemic hemodynamic parameters were assessed by PiCCo monitoring in both groups.

**Results:**

Resting hemodynamic parameters were identical in the two groups. *In control animals*, systemic hemodynamic variables were relatively stable during the entire procedure. *In septic animals* shock developed in 165 (IQR: 60–255) min after starting the injection of *E. coli* solution. Blood pressure values gradually decreased, whereas pulse rate increased. A decrease in cardiac index, an increased systemic vascular resistance, and a decreased stroke volume variation were observed.

**Conclusions:**

These results may serve as additional pathophysiological information of hemodynamic changes occurring during hypodynamic sepsis and may contribute to a better understanding of the pathomechanism of septic multiple organ failure.

## Background

Sepsis is a serious condition characterized by systemic response of the inflammatory and coagulation system. The generalized response of the body to the infectious agent is overwhelming, leading to hemodynamic deterioration, capillary leak, increased coagulation, disturbances of organ perfusion and multiple organ failure. Despite the extensive clinical and experimental research, sepsis remains a major cause of morbidity and mortality [1].

Different animal models have been used so far for better understanding sepsis pathophysiology and for testing new treatment strategies. Among others, endotoxemia models, bacterial infusion models, cecal ligation and puncture models, and colon ascendent stent peritonitis models have been most frequently applied for the assessment of septic process under experimental conditions. A common feature of endotoxicosis and bacterial infusion models is that they are controlled and standardized. Their drawback, however, is the lack of infectious focus and the rapid onset of hemodynamic response as compared to human sepsis [2]. Despite these limitations, in the recent decade intravascular infusion of living *E. coli* or *Pseudomonas aeruginosa* has been frequently used in baboons, dogs and porcine models for the assessment of the immunological, hemodynamic and metabolic processes [3, 4]. It has been reported that intravenous infusion of bacterial cultures results in a rapidly developing hypodynamic septic shock.

In human sepsis, pulse-induced contour cardiac output (PiCCO) monitoring is the key method of hemodynamic monitoring and contributes to guidance of treatment strategies [5]. Unfortunately, in porcine models hemodynamic data using the PiCCO monitor system are scarce. In view of this, the aim of the present work was to describe the systemic hemodynamic response in a porcine model of *E. coli*-induced sepsis model. The present work is the first part of a complex study plan that aimed to assess the hemostatic, hemorheological and cerebrovascular consequences.

## Materials and methods

### Experimental animals and protocol

Thirty-one healthy female Hungahib pigs, 10–12 weeks old, were randomly assigned into two groups: *control* (*n* = 15) or *septic group* (*n* = 16). The weight and length of the animals in the two groups were: weight, control 18.9 (16.7–20.4) kg vs. weight, septic: 18.9 (18.8–20.6) kg, *p* = 1.0; length, control: 92 (83.5–97.2) cm vs. length, septic: 92 (85–98.2) cm, *p* = 0.96. According to the original protocol the inclusion of 32 animals was planned (16 pigs in each group).

The experiments were approved and registered by the University of Debrecen Committee of Animal Welfare (Permission Nr.: 21/2013. UD CAW), in accordance with the Hungarian Animal Protection Act Law XVIII/1998 and the Ordinance 40/2013. (II.14.) of the Hungarian Government and EU directive (2010/63/EU).

In the *sepsis group, Escherichia coli* culture (2.5 × 10^5^/ml; strain: ATCC 25922, Department of Medical Microbiology, University of Debrecen) suspended in physiological saline (Api NaCl 0.85 Medium and suspension medium, bioMérieux SA, Lyon, France) was intravenously administrated in a continuously increasing manner according to the following protocol: 2 ml of bacterial culture suspended in physiological saline was injected in the first 30 min, then 4 ml of bacterial culture was administered within 30 min, followed by infusion of 32 ml bacterial culture for 2 h. Thus, a total of 9.5 × 10^6^ *E. coli* was administered within 3 h. According to our laboratory tests, at 3 h after suspending the *E. coli*, the number of living bacteria remained stable.

Subjects of the *sepsis group* were examined until they died as a cause of the fatal infection. In the *control group* infusion was administered in a similar volume to the *septic group* of isotonic saline solution and no further intervention was carried out on them. Each individual of this group was followed for 8 h (if the animals had not died earlier), and at the end of the experimental period the animals were over-anesthetized.

The study was carried out under general anesthesia maintained by giving intramuscular ketamine (15 mg/kg) and xylazine (1 mg/kg) throughout the experiment. Anesthesia was guided according to blood pressure and heart rate changes to noxious stimuli, and was adjusted if necessary by intermittent boluses of ketamine and xylazine. Both in the *sepsis* and *control* groups, inferior tracheostomy was performed and an endotracheal tube was inserted for supported ventilation. Pressure support mechanical ventilation (Airox Legendair Ventilator, PAU CedexFrance) was used. Mechanical ventilation was adjusted to secure a PaO_2_ of 100–130 mmHg and PaCO_2_ of 35–45 mmHg.

Besides physiological saline infusion, the animals were not given anticoagulants, further intravenous volume replacement or any further medication during the experiment. The temperature of the operating room was set to approximately 25 °C and a 37 °C heating pad was placed under the animals to maintain body core temperature above 37 °C. A suprapubic cystostomy catheter was placed to ensure urinary drainage.

### Hemodynamic measurements

The left external jugular vein and the left femoral artery were surgically prepared and cannulated for invasive hemodynamic measurements, and blood sampling. After all surgical interventions had been completed, a 1-h-long stabilization period was allowed before the beginning of the experimental protocol. Systemic hemodynamic variables were assessed by thermodilution using a 4F, 8 cm PiCCO^®^-Catheter (Pulsion Medical Systems AG, Munich, Germany) with the injection of 10(×) ml of cold saline each hour. Heart rate [HR (1/min)] and mean arterial pressure [MAP (mmHg)] were monitored invasively through the femoral artery catheter. The Meeh’s formula was used for calculation of body surface area in pigs (BSA = 8.58 × BW).

### Sampling

Measurements were performed at resting state = TR, and every hour after starting of the injection of *E. coli* or isotonic saline during the experiment. Thus, data from T60 to T360 were registered and are presented. Resting measurements were performed before starting suspension or saline (indicated as RS). At 60 min (indicated as T60), the injection of 2 + 4 ml bacterial culture/saline was completed, at 120 min (indicated as T120) and at 180 min (T180) additional 16 ml cultures or saline solutions were infused.

### Statistical analysis

Statistical analysis was performed using SPSS 19.0 (SPSS, Chicago, IL). Kolmogorov–Smirnov test was used to verify the normality of the distribution of continuous variables. As the majority of the parameters did not show normal distributions, data are presented as medians and interquartile ranges and parameters were compared by the appropriate non-parametric tests (Mann–Whitney rank sum test).

The analysis of the treatment effect on the different parameters within the groups occurred by using repeated measures ANOVA. To calculate the correlation between different variables Spearman correlation was used. A *p* < 0.05 was defined as statistically significant difference.

## Results

Of the 16 septic animals, 2 died after 2 h, another 5 at 3 h, another 3 at 4 h, another 4 at 5 h and 1 at 6 h after the *E. coli* infusion was started.

*Resting systemic hemodynamic parameters* of the control and septic groups are summarized in Table 1. Hemodynamic parameters were similar in both groups before starting the injection of the *E. coli* suspension.

**Table 1:** Systemic hemodynamic parameters at resting state in control and septic animals

Parameters	Control	Septic	*p* value
MAP (mmHg)	102 96.5–110.7	106 97–111.5	0.86
Pulse rate (1/min)	92 81.5–102.5	99.0 81.7–108.5	0.50
CI (l/min/m^2^)	2.8 1.8–2.9	2.9 2.1–3.2	0.08
SVRI (dyn s cm^−5^ m^2^)	2855 2452–3125	2600 2214–2719	0.06
GEDI (ml/m^2^)	411 353–520	538 414–631	0.02
SVV (%)	13 11–17	13 8–16	0.52
GEF (%)	29 23–34	23 20–27	0.12
EVLWI (ml/kg)	13 9–16	17 12–21	0.08

Medians and interquartile ranges are presented

*In control animals*, hemodynamic variables were relatively stable during the entire procedure. Mean arterial pressure decreased at 6 h and systemic vascular resistance index increased from 5 h on (Table 2).

**Table 2:** Systemic hemodynamic parameters measured in control animals

Parameters	Time point of measurement							
	TR (*n* = 15)	T60 (*n* = 15)	T120 (*n* = 15)	T180 (*n* = 15)	T240 (*n* = 15)	T300 (*n* = 6)	T360 (*n* = 6)	*p* value
MAP (mmHg)	102 96.5–110.7	103 92–108.7	96 87–109.7	100 89–110	99 85.2–109	102 93–103	92 80–99	< 0.001
Pulse rate (1/min)	92 81.5–102	94 77–103	88 83–103	94 85–97	88 81.5–95.5	87.5 82–96	83.5 81–87	0.98
CI (l/min/m^2^)	2.27 1.8–2.9	2.4 1.6–2.8	2.2 1.4–2.6	2.3 1.6–2.6	2.0 1.7–2.7	1.8 1.6–2.5	1.9 1.5–2.1	0.18
SVRI (dyn s cm^−5^ m^2^)	2855 2452–3125	2991 2771–3291	3238 2906–3867	3138 2510–4040	3113 2799–4450	4454 3388–4700	4054 3657–4740	< 0.01
GEDI (ml/m^2^)	411 353–520	414 320–479	389 308–465	395 305–484	373 312–447	230 181–350	207 188–350	0.37
SVV (%)	13 11–17	15 11–18	15 11–19	15.5 11–19	14 9.7–20	16.5 12–19	17 14–18	0.79
GEF (%)	29 23–34	27.5 23–34	27 21.2–28	26 23–28	26 23–29	26 25–28	26 24–29	0.02
EVLWI (ml/kg)	13 9.6–16.7	14 9.2–18	16 8.5–19	15 7.7–18.2	13.5 8–17	9.5 6–13	10 6–14	0.07

Medians and IQRs are presented. TR = resting state, T60–T360 = minutes after starting the injection of the *E. coli* suspension

**Fig. 1:**
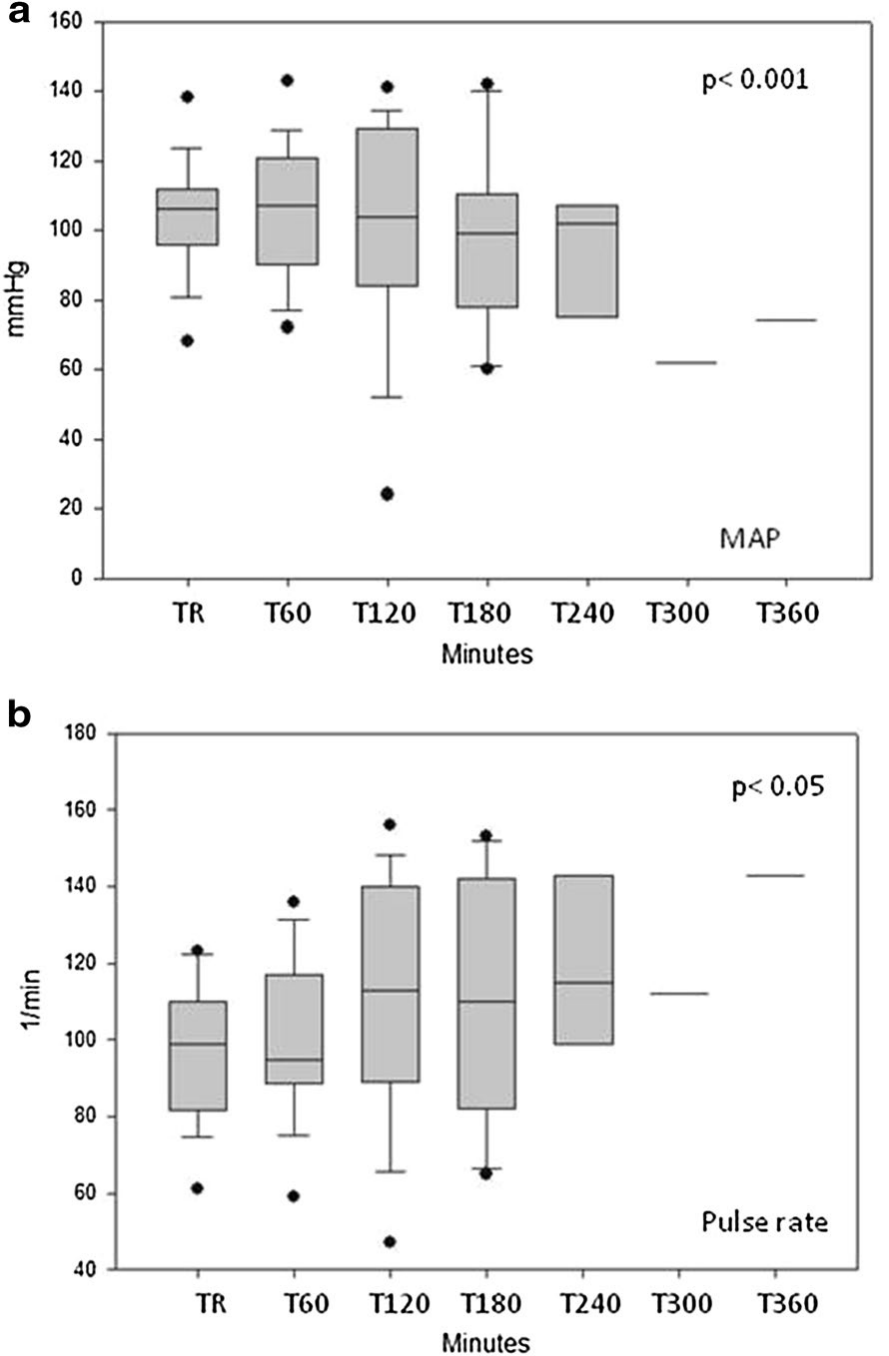
Mean arterial pressure and pulse rate in septic animals during the experiments (medians and interquartile rages are presented). TR: resting state, T60–360 = time after starting the *E. coli* infusion in minutes

**Fig. 2:**
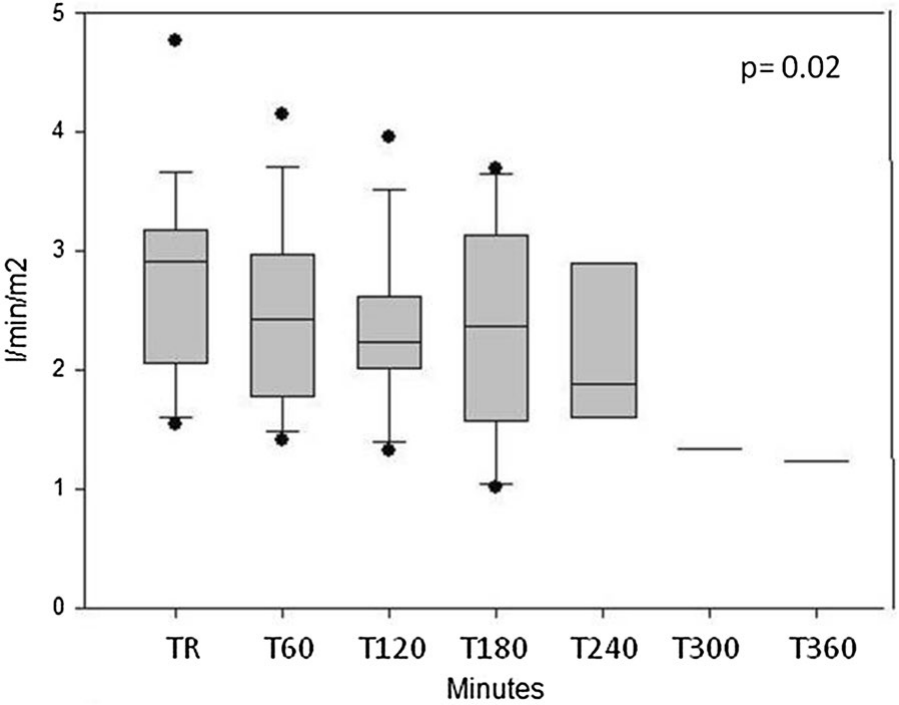
Cardiac index values in septic animals during the experiments. (medians and interquartile rages are presented). TR: resting state, T60–360 = time after starting the *E. coli* infusion in minutes

**Fig. 3:**
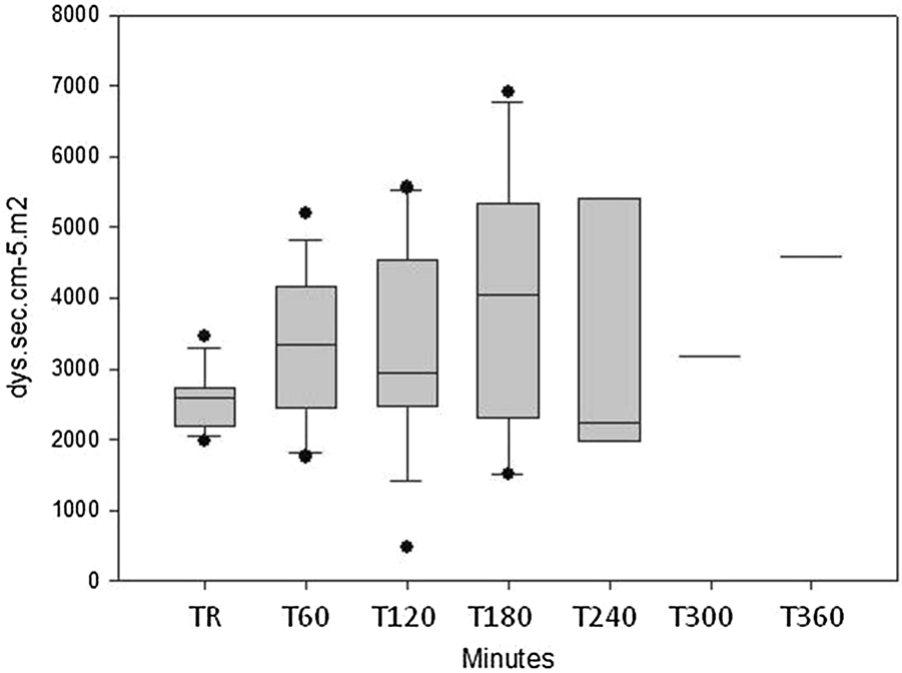
Changes in systemic vascular resistance index in septic animals. (medians and interquartile rages are presented). TR: resting state, T60–360 = time after starting the *E. coli* infusion in minutes

**Fig. 4:**
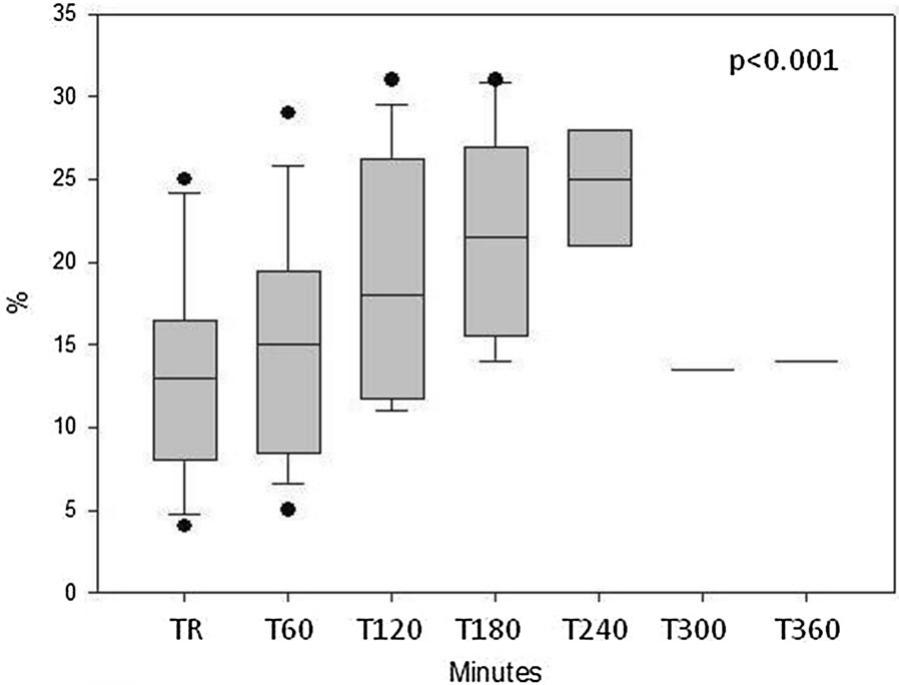
Changes in stroke volume variation in septic animals. (medians and interquartile rages are presented). TR: resting state, T60–360 = time after starting the *E. coli* infusion in minutes

*In septic animals*, shock developed in 176 (IQR: 60–263) min after starting the injection of *E. coli* solution. The change in the different parameters in this animal group is summarized in Table 3. Blood pressure values gradually decreased, whereas pulse rate increased in the pigs after injection of *E. coli* (Fig. 1a, b). Flow parameters indicated a decrease in cardiac index (Fig. 2). Systemic vascular resistance (SVRI) showed a temporary increase at 3 h, but returned to the initial value thereafter (Fig. 3). Additionally, stroke volume variation (SVV) showed a statistically significant change (Fig. 4) with a non-significant decrease in global end-diastolic index (volume parameters). Extravascular lung water index generally increased, while global ejection fraction decreased during the procedure (Table 3).

**Table 3:** Systemic measured in septic animals

Parameters	Time point of measurement							
	TR (*n* = 16)	T60 (*n* = 16)	T120 (*n* = 14)	T180 (*n* = 9)	T240 (*n* = 6)	T300 (*n* = 2)	T360 (*n* = 1)	*p* value
MAP (mmHg)	106 97–111.5	107 90.5–119.7	104 84.7–127.7	99 81–107	102 75.2–106	62 50–74	74	< 0.001
Pulse rate (1/min)	99 81.7–108.5	95 89.7–115.5	113 89.2–138.7	110 83–142	115 102.5–141	112 109–115	143	0.05
CI (l/min/m^2^)	2.91 2.1–3.2	2.4 1.8–2.9	2.2 2.0–2.6	2.4 1.7–3.1	1.9 1.6–2.8	1.3 1.0–1.7	1.2	0.02
SVRI (dyn s cm^−5^ m^2^)	2600 2214–2719	3350 2497–4010	2952 2495–4473	4047 2541–5220	2243 2047–5370	3182 677–5682	4585	0.162
GEDI (ml/m^2^)	538 414–631	491 409–601	491 415–604	482 414–548	491 394–582	200 147–254	138	0.535
SVV (%)	13 8–16	15 9–19	18 12–26	21.5 16–26	25 16–27	13.5 13–14	14	< 0.001
GEF (%)	23 20–27	21 19–26	19 18–25	20 15–23	21 16–25	20 19–22	19	< 0.001
EVLWI (ml/kg)	17 12–21	19 12–23	19.5 15–25	18.5 12–22	18 8–17	9.5 13–21	5	0.03

Medians and IQRs are presented. TR = resting state, T60–T420 = minutes after starting the injection of the *E. coli* suspension

## Discussion

In the present experimental study, we used bacterial infusion model for assessing hemodynamic consequences. It has been demonstrated in previous studies that intravenous infusion of *E. coli* results is an increased production of pro-inflammatory cytokines leading to a hypodynamic circulatory response of the host organism. This is the first study using the PiCCO system for monitoring the systemic hemodynamic changes in *E. coli*-induced intravenous experimental sepsis model without any therapeutic intervention. In a different porcine sepsis model, using the cecal ligation peritonitis method, Schuerholz et al. [6] already proved the usefulness of the pulse contour continuous cardiac output monitoring. Barrett-Due et al. [7] reported on a decreased mean arterial pressure, increased pulse rate and decreased systemic vascular resistance after infusion of *E. coli* suspension. Using the Swan–Ganz catheter technique, Pranskunas et al. [8] reported on a cardiac index that decreased by half and a systemic vascular resistance that doubled after intravenous infusion of *E. coli* suspension.

Our results are fairly comparable with these previous porcine experiments: in septic animals, we observed a decrease in mean arterial pressure and cardiac index along with an increased pulse rate during development of septic shock. An additional finding of the present study is the significant change of the stroke volume variation in septic animals. It has to be noted that in our study fluid administration was not used during the experiments, because we intended to monitor the hemodynamic changes without any therapeutic interventions. This is the explanation why a slight, but statistically significant change was observed in the mean arterial pressure and the systemic vascular resistance of control animals. When analyzing the mean arterial pressure values in control animals—although the changes were statistically significant—MAP values remained within a relatively stable, clinically acceptable limits ranging from 96 to 103 mmHg. The fact that cardiac index remained stable during the experiments in control animals excludes the cardiodepressive effect of xylazine and ketamine that were used for general anesthesia.

Intravenous infusion of living bacteria—such as *E. coli*—results in cardiovascular collapse and early death in experimental animals. Low cardiac output, deterioration of the cardiovascular system and organ hypoperfusion have been documented in baboons, dogs and in porcine models [4]. This hypodynamic response may be the result of the overwhelming production of TNF-alpha and IL-1beta cytokines. A relationship has been documented between the circulating levels of these cytokines as well as the development of septic hypodynamic response and shock [9]. Additionally, antibodies against these pro-inflammatory factors were capable of preventing the development of septic shock in animal experiments [10, 11].

Obviously, the development of septic shock after intravenous infusion of living *E. coli* is different from other experimental sepsis models [1, 4]. Other sepsis models are characterized by a biphasic response starting with a hyperdynamic phase (increased cardiac index and cardiac output, low mean arterial pressure and low peripheral vascular resistance), followed by a hypodynamic phase (decreased cardiac output and systemic vascular resistance); therefore, the present results are less comparable with them due to the obvious methodological differences. It seems that the inflammatory response that is evoked by *E. coli* infusion is directed toward the cardiac contractile function (both cardiac index and global ejection faction decreased during the process). As a consequence of this hypodynamic circulatory response, an immediate microcirculatory impairment was observed in an early phase of the bacterial injection [12], whereas cerebral autoregulation remained unaffected [13]. It has been also documented that due to this hemodynamic collapse, lactate levels got elevated followed by the increased appearance of nucleated red blood cells, indicating tissue hypoxia [14]. Thus, intravenous infusion of *E. coli* results in a fulminant hypodynamic sepsis leading to tissue hypoperfusion and hypoxia.

We have to mention the limitations of our study. First, due to the high mortality in the septic group due to fulminant hypodynamic sepsis during the early hours of the experiment, the results are less comparable to the control group. This is the explanation why we mainly focused on the description of the group characteristics of the hemodynamic responses. Another important limitation of the present study is the lack of fluid resuscitation during the experiments. However, while planning the study we intentionally decided to not use fluid resuscitation, because we wanted to test the natural history of *E. coli*-induced sepsis. Other porcine experiments using fluid resuscitation strategy and hemodynamic monitoring reported on similar results despite fluid administration [6, 8].

In conclusion, in the present study, we described the hemodynamic response to intravenous injection of *E. coli* in pigs using the PiCCO monitoring. Despite the limitations and criticisms of this animal model, the results may be useful for better understanding of the natural history of certain extreme clinical sepsis scenarios such us meningococcemia, pneumococcus-related or Gram-negative sepsis developing in granulocytopenic patients and may help in the development of effective treatment strategies. It has to be noted, however, that the findings observed in *E. coli* (Gram negative)-induced sepsis cannot be generalized to sepsis due to other Gram-positive bacteria.
